# U-Hack Med Gap Year—A Virtual Undergraduate Internship Program in Computer-Assisted Healthcare and Biomedical Research

**DOI:** 10.3389/fbinf.2021.727066

**Published:** 2021-10-11

**Authors:** Stephan Daetwyler, Hanieh Mazloom-Farsibaf, Gaudenz Danuser, Rebekah Craig

**Affiliations:** Lyda Hill Department of Bioinformatics, UT Southwestern Medical Center, Dallas, TX, United States

**Keywords:** bioinformatics, science education, undergraduate research, virtual experience, peer-mentoring

## Abstract

The COVID-19 healthcare crisis dramatically changed educational opportunities for undergraduate students. To overcome the lack of exposure to lab research and provide an alternative to cancelled classes and online lectures, the Lyda Hill Department of Bioinformatics at UT Southwestern Medical Center established an innovative, fully remote and paid “U-Hack Med Gap Year” internship program. At the core of the internship program were dedicated biomedical research projects spanning nine months in fields as diverse as computational microscopy, bioimage analysis, genome sequence analysis and establishment of a surgical skill analysis platform. To complement the project work, a biweekly Gap Year lab meeting was devised with opportunities to develop important skills in presenting, data sharing and analysis of new research. Despite a challenging year, all selected students completed the full internship period and over 30% will continue their project remotely after the end of the program.

## Introduction

The COVID-19 health crisis has heavily impacted traditional learning environments (Slavin and Nathan, 2020; UNESCO 2020). Many universities have cancelled or shifted their courses to a virtual learning space, depriving students of essential elements of the college experience (Abbasi et al., 2020; Kogan et al., 2020; Sidpra et al., 2020). This includes laboratory rotations, hands-on-experiments, face-to-face social interactions and important opportunities to learn together and discuss assignments (Daniel 2020; Kogan et al., 2020). Consequently, in the 2020–2021 academic year many students decided to take a “gap year” and were looking for new opportunities to be involved in active research.

The Lyda Hill Department of Bioinformatics at UT Southwestern Medical Center (UT Southwestern) was also affected in educational programming for undergraduates. Traditionally, every year UT Southwestern offers an international hackathon for students interested in application of computational approaches to solve “real-world” biomedical problems (www.u-hackmed.org). These events take place in-person, across 3 days during which teams of students, researchers and physicians intensely endeavor to bring new computational approaches to clinical and biomedical research and utilize the analytic power of the BioHPC, UT Southwestern’s high-performance computing resource. The hackathon format attracts students from community colleges to Ivy League schools and is particularly successful, as students work on clearly pre-defined, stimulating research questions with faculty across UT Southwestern. Unfortunately, during the COVID-19 health crisis, travel and laboratory restrictions led to the cancellation of this successful format.

**FIGURE 1:**
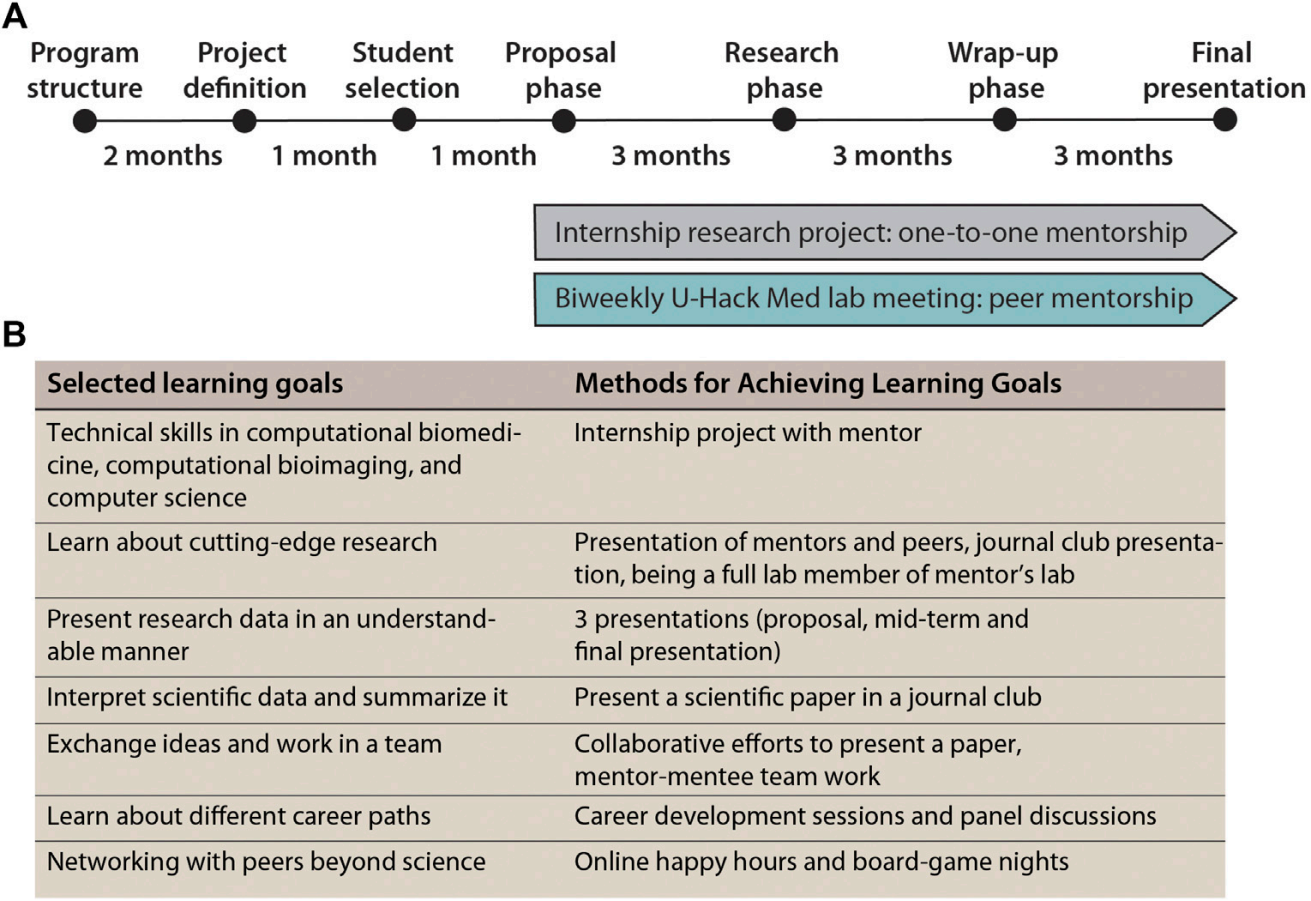
Project milestones and learning goals. **(A)** Schematic of important milestones of the U-Hack Gap Year internship program **(B)** Summary of the major learning goals for the undergraduate students.

To overcome this lack of exposure for undergraduate students to pressing questions in healthcare and computer-assisted biomedical research, a new format, the U-Hack Med Gap Year program was conceived. To comply with local and state regulations, the internship was defined as a completely remote, virtual experience that provided new opportunities for industrious students searching for meaningful research projects during this time of crisis. At the core of our U-Hack Med Gap Year internships were clearly defined projects that were attractive to undergraduate students, useful for mentors to advance their research program, and discrete in scope so that substantial project completion could be achieved within 6–9 months. To build a sense of community among the intern cohort and mentors, the experience was enhanced with a biweekly virtual U-Hack Med lab meeting. In the next sections, we describe administrative best practices for setting up a virtual internship program, the project and student selection process, the internship program structure with an initial proposal phase, a core research phase, and a wrap-up phase with a student symposium, before describing in detail the surveyed outcome of the program (Figure 1).

### Administrative Structure for Remote Internship Program

Offering innovative opportunities such as a fully remote, paid internship may require overcoming administrative barriers as a first step. Many institutions of higher education have not traditionally allowed fully remote work due to university telecommuting policies, tax-withholding issues, and other state regulations. The COVID-19 crisis presented a window of opportunity for advocacy toward greater openness and administrative restructuring with regard to remote work. Prior to launching the U-Hack Med Gap Year applications, facilitators coordinated with several administrative offices including graduate school, payroll, accounting, international affairs and human resources temporary employment recruitment. From these meetings, emerged the following recommendations for how research departments in the United States can administratively structure this type of program.

*State-to-state registration*: To employ remote workers in another state, employers must register with the department of state in that jurisdiction and these requirements vary by state. It is important to determine what states the institution is registered, or is willing to register in, prior to selection of student applicants.

*Job classification*: A custom job code, reserved for undergraduate students, was used for this opportunity. The job code allowed the intern to remain in “student” status with compensation in the form of a monthly stipend versus being considered a “temporary staff” employee paid an hourly wage. This distinction is important as stipends support educational and training opportunities and thus do not represent compensation for work performed. Depending on the job code used, stipends therefore may not be subject to the federal and state payroll taxes that are assessed for salaries and wages. This is an important factor to be considered, together with the states an employer is registered in, to determine the geographic region from which remote student interns may be recruited.

*Student stipend:* To support living expenses during the course of the internship, every accepted student received a stipend of $1,600 dollars per month. To incentivize research labs to participate in the program, the Department subsidized 50% of the stipend cost for each student, while the sponsoring lab was responsible for providing the remaining 50% of the funding.

*Remote employee onboarding*: It is important that a system is established for onboarding of a fully remote employee. UT Southwestern requires in-person “check-in” for all employees during their first 3 days of employment. During this appointment, personal documents are verified, and an I-9 employment eligibility verification form is completed by the human resources representative. UT Southwestern partnered with a third-party vendor at a nominal fee per student. Each student intern was then able to complete the check-in with this authorized representative at a location within driving distance of their place of residence. Another aspect of remote employee onboarding made possible in response to the COVID-19 crisis was the institution’s mandatory new employee orientation. This typically is required as an in-person group meeting on the first day of employment. However, the human resources department quickly adapted to the need for social distancing by moving all new employee orientation sessions to a virtual platform.

*International student interns*: The international affairs office of each institution has some measure of discretion in interpretation of federal regulations. In the UT Southwestern context, completion of the I-9 employment eligibility verification by a third-party vendor for international students was not allowed. Since travel and on-campus activity was also severely limited by the COVID-19 crisis, it was determined that students in a temporary visa status could not be admitted to the program. This was made clear in the application announcement.

### Project Selection and Scope

**FIGURE 2:**
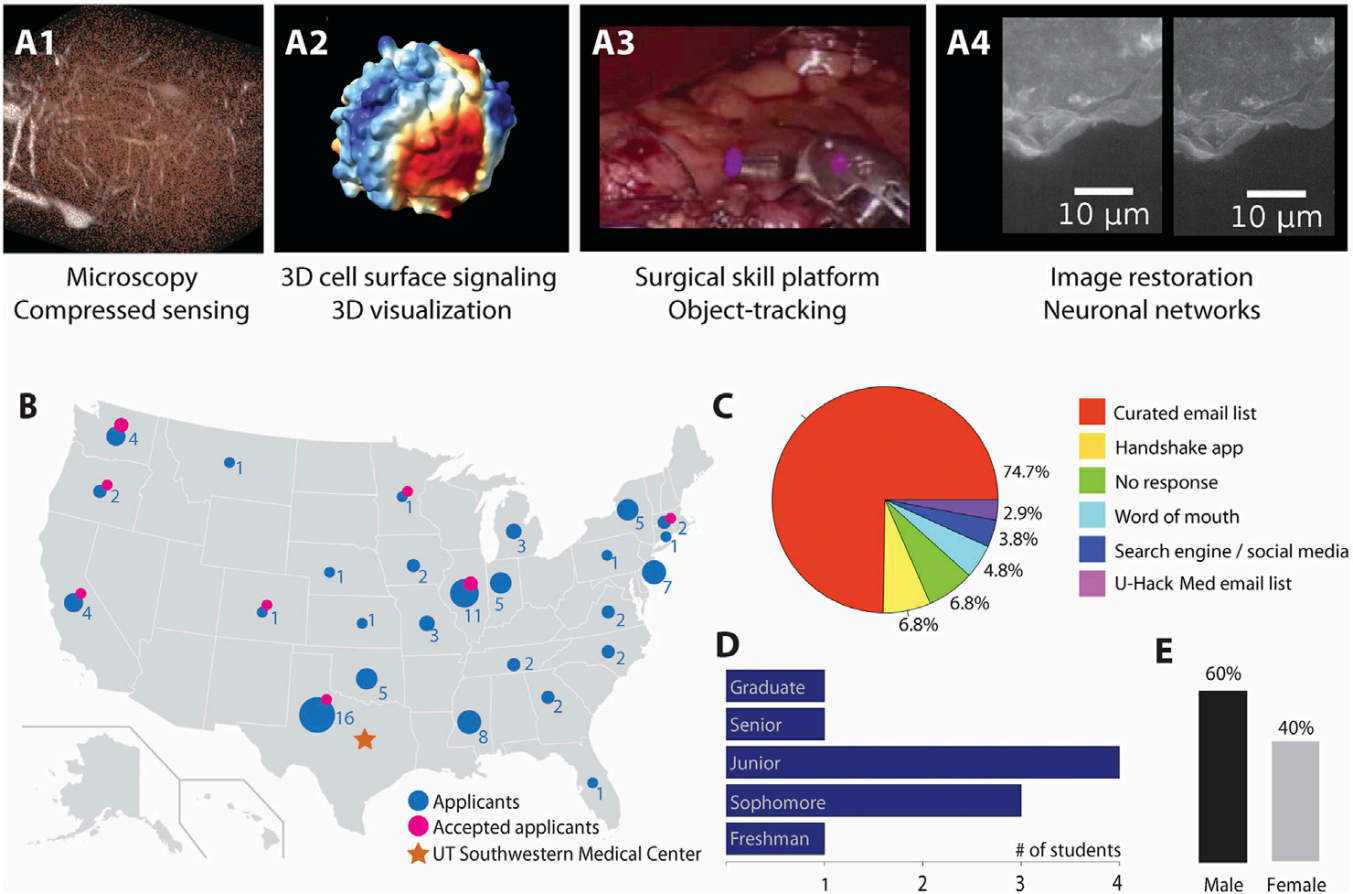
Internship projects and student characteristics. **(A1-A4)** The internship projects spanned topics as diverse as microscopy, 3D visualization, surgical skill platform analysis and image restoration. **(A1)** A student applied compressed sensing algorithms (Rani, Dhok, and Deshmukh 2018) to reconstruct an original image of mouse brain neurons (white) from sparsely sampled points (brown) in a 3D volume obtained by simulating a random-access microscope. **(A2)** A student analyzed molecular signals on a 3D cell surface of a melanoma cell (Driscoll et al., 2019) and visualized the resulting data. **(A3)** A student established a full pipeline to perform automated analysis to score the performance of different surgeons on a novel surgical skill platform (Battaglia et al., 2021). **(A4)** A student explored artificial neuronal networks such as CARE (Weigert et al., 2018), to obtain higher resolution images (right) from raw data (left). **(B)** Applications (blue, circle size scaled for number of applications) for the U-Hack Gap Year were received from all over the US. Consequently, participating students (pink, circle size scaled for number of interns) were working remotely from all over the US, while the mentors were working at UT Southwestern in Dallas (orange). **(C)** Source of information that made participant aware of the U-Hack Med Internship program included advertisement through our curated college email list (74.7%), Handshake app (6.8%), no response (6.8%), word of mouth/friend (4.8%), search engine and social media (3.8%), U-Hack Med former participant mailing list (2.9%). **(D)** The knowledge background of the participating students was highly diverse ranging from freshman to graduate student knowledge. **(E)** The internship participants were six male and 4 female students.

To capture the breadth of research within the Lyda Hill Department of Bioinformatics, all faculty were invited to draft project proposals and present their ideas in a planning meeting. Ideal projects were limited in scope so that they could be completed in 6–9 months with a student time commitment of approximately 30 h per week. Since the interns would be fully remote, projects also had to be feasible without any specialized equipment. In total, nine faculty members proposed eleven projects and half of the faculty integrated trainees and senior researchers from their labs as project leaders and mentors. All eleven projects complied with the requirements and were selected for publication on the U-Hack Med website (www.u-hackmed.org/2020gapyear). The projects were as diverse as visualization of 3D data, image processing with deconvolution, genome sequencing with deep-learning networks, and developing an automated surgical skill analysis platform (Figure 2A; Table 1).

**TABLE 1:** Overview about selected projects. Graduate Student (GS), Postdoctoral Fellow (PF), Instructor (I), Principal Investigator (PI). * Biweekly lab meeting coordinator.

Mentor(s)	Project title
J. Zhou (PI)	Making Deep Learning Models Interactive and Self-Interpreting
X. Wang (PF), G. Danuser (PI)	Building a Deep Learning-Based Shape Selector
H. Mazloom-Farsibaf* (PF), M. Driscoll (I), G. Danuser (PI)	Computer Graphics of Cancer Cells
B.J. Chang (I), R. Fiolka (PI)	Deep Learning Deconvolution
S. Daetwyler (PF), R. Fiolka (PI)	Really Smart Microscopes for Cancer Cell Biology
J. Lee (PI)	Deciphering Visual Evaluation on Reconstructive Surgery Outcomes Using an Eye-tracking Platform and Machine Learning Techniques
A. Jamieson (PI)	Developing an Automated Surgical Skill Analysis Platform
S. Nguyen (PF), A. Treacher (GS), A. Montillo (PI)	Image Segmentation, Deep Learning Architectures & Predictive Modeling to Advance Neuroscience
D. Beshnova (PF), Bo Li (PI)	Interpretable Deep Learning for Cancer-Associated T-Cell Receptors
D. Kim (PI)	3D Visualization of An Entire Cell
S. Rajaram (PI)	Deep Learning for Histopathology and Broad Utility Biomedical Tools

### Student Recruitment

To recruit students, we leveraged a curated list of administrator contacts in STEM departments at colleges and universities across the US. This list was first established for the U-Hack Med Hackathon, with the help of high school interns who personally contacted hundreds of university administrators to introduce the U-Hack Med program and invite their partnership in promoting the event. For the U-Hack Med Gap Year promotion, university career services or advising office contacts were added. While initially a labor-intensive task, this list is now updated annually and has proven effective in widely promoting the U-Hack Med events. Through this list, a promotion email for the 2020 Gap Year program was sent to 808 administrators at 132 research-intensive undergraduate universities. Besides this list, the UT Southwestern and U-Hack Med websites, social media networks, email to former U-Hack Med hackathon participants, and the student recruitment platform *Handshake* were used to advertise. From the 133 student applicants, we learned that emails directly to university STEM departments and career offices were the most effective form of promotion, with nearly 75% of applicants indicating this communication as the way they learned about the program (Figures 2B,C).

To apply, undergraduate students were required to submit a resume, transcript and personal statement. In the personal statement, applicants were asked to describe their educational and career goals, circumstances that led to interest in a gap year internship, and their motivation for a specific U-Hack Med Gap Year project. In the resume, students were further asked to include links to code samples and completed or in-process computational or research projects.

Each mentor was asked to review applications and rank their preferred interns with consideration for the intern’s project preferences. In a group meeting of all mentors, possible matches were tallied and from an initial 133 applicants, 26 students were selected for interviews. Group interviews were arranged with all potential intern-mentor pairs to determine the best fit of intern skills and interest with project requirements. They also served to further narrow the pool of applicants. Final assignments were made in a subsequent mentor meeting. For one of the projects, there was only one suitable student applicant, but the selection process helped to determine that another project was a better fit for that student’s interests and enthusiasm; thus, the faculty member chose to withdraw the project from consideration. A final ten matched students were then invited to participate.

The selection process led to a student population with diverse knowledge backgrounds (Figure 2D). Most students were junior classification (40%), followed by sophomore (30%), while freshmen, senior and recent graduates each represented 10% of the intern cohort. Moreover, the interns were located all over the US and had a gender distribution of 40% female and 60% male (Figure 2E). Of note, this percentage of female students is significantly higher than the percentage of women in computer science [19% on Bachelor’s level, (NSF 2017)].

### Key Features of the U-Hack Med Gap Year Program

At the heart of the U-Hack Med Gap Year program was a dedicated research project. To achieve project goals, students received one-to-one mentoring and participated as full lab members in the mentor’s lab. This included virtually attending all lab meetings. In several cases, graduate students and postdoctoral fellows were paired with interns (Table 1) to achieve defined research goals as a mentee-mentor team, providing an excellent professional growth opportunity for both students and mentors. In these cases, an experienced faculty member further supervised the mentee-mentor team.

As many projects were computationally expensive, UT Southwestern provided all students access to its Biomedical High-Performance Computing cluster, the BioHPC, via remote VPN login. The BioHPC provides state-of-the art service with access to over 30 PB of data storage capacity and 28,000 CPU cores (https://portal.biohpc.swmed.edu/content/). Currently, the simultaneous allocation limit for an individual user (and thus the interns) is set at 4 GPU nodes with high-end Nvidia GPU modules (Nvidia Tesla K20/K40, P4, P40, P100, V100, A100) and 64 “light-weight” CPUs nodes with each 32 GB memory, or alternatively 16 large memory nodes with up to 384 GB memory. To further facilitate computational analysis, the BioHPC is equipped with remote virtual desktops to access the cluster and licensed software needed for the projects. As part of the onboarding process, all students received training to access and use the compute cluster for their analysis.

Besides the research project, the biweekly U-Hack Med lab meeting was a crucial element of the U-Hack Med Gap Year internship program. It provided a platform to achieve important learning goals (Figure 1B). As students came with diverse academic backgrounds (Figure 2D), the lab meeting included high-level training in presentation, interpreting research data, structuring data and data sharing. Specific technical training was the responsibility of the mentors. A postdoctoral fellow was chosen to design and lead the biweekly program and student symposium, with the input and mentoring of two faculty members, providing additional trainee professional enrichment and growth.

The structure of the U-Hack Med lab meetings supported their internship experience. First, to give students time to become fully acquainted with their project, all mentors presented their work to highlight ongoing cutting-edge research at UT Southwestern. Mentor talks were complemented with toolbox presentations on how to give a presentation. This phase was concluded with a first “proposal” presentation by the students. This proposal presentation was intended for students to concisely articulate their ideas and understanding of the project and receive feedback. Afterwards, students worked on their projects and showcased their progress in a mid-term presentation. Alongside these, the students teamed up in pairs to choose a new research paper and present it in a journal club format. The journal club presentation was a central element in the training effort to enhance the students’ skills in extracting the main messages of a paper. During the wrap-up phase, two career sessions were included to increase students’ awareness of job opportunities for biomedical scientists. One session was dedicated to industry careers with an invited speaker. The second session was a panel discussion devoted to academic careers with two faculty and two trainees from the Lyda Hill Department of Bioinformatics at UT Southwestern. Finally, the U-Hack Med lab meetings culminated in a symposium with a presentation by each student and attendance from the broader Department and institutional education leaders.

The internship experience was further augmented by optional community building events. These included two “Virtual Happy Hour” sessions, where internship students and mentors met in a relaxed and friendly atmosphere to celebrate holidays such as the New Year. In addition, two virtual board game nights were organized with group games such as Pictionary and Code Names.

### Outcome

Despite the numerous challenges during an unprecedented international crisis, all ten selected students completed the program. Students were successful in developing computer vision pipelines for performing automated analysis on raw video data from actual surgical procedures, deep learning-based object tracking, establishing of containers for high-performance cluster computation, image reconstructions from sparse signals, deconvolution of imaging data, restoration of low-resolution microscopy images to high-resolution images at a tenfold speed increase compared to conventional methods, building of a novel application of artificial intelligence to clinical image segmentation, and molecular signal analysis on 3D cell surfaces. Three of the ten students have already decided to continue their research project. At least eight of the ten projects have publications in planning or close to submission.

To ascertain areas in which the U-Hack Med Gap Year program was successful and could improve, we developed anonymous online surveys completed by all ten students and eight mentors (Supplementary Notes 1 and 2). As illustrated in Supplementary Figures S1, S5, students found the application process user-friendly (80 ± 13%). Mentors (79 ± 5%) and students (90 ± 8%) agreed that the matching process was also satisfactory. Students felt somewhat well prepared by their academic program for the internship (76 ± 10%), while mentors expressed a lower confidence in the academic preparation of interns (63 ± 18%). Students and mentors shared in open-ended comments that a better and longer training was needed at the beginning of the internship for using high-performance cluster computing resources.

During the biweekly lab meetings (Supplementary Figures S2, S5), students gained the most benefit from preparing and presenting their research in the final student symposium (83 ± 17%), a career session on prospects in industry (78 ± 11%), learning about the other projects (77 ± 13%), and the panel discussion on careers in academia (70 ± 13%). Moreover, mentors appreciated the biweekly lab meetings and felt it brought good benefit to the students (84 ± 14%). Open-ended comments from both students and mentors indicated opportunity for improvement in the amount of constructive feedback offered by other interns and mentors during biweekly meetings, and in the use of these meetings to develop problem-solving skills.

Overall, students perceived communication with the mentors via virtual platforms (Supplementary Figure S3) as “just right” and felt communication was frequent and easy enough (92 ± 8%). Mentors agreed that communication was frequent and easy in a completely virtual environment (81 ± 13%). Approximately half of the mentors (46 ± 9%) and students (40 ± 11%) felt that the project period was long enough for the students to make satisfactory progress. However, mentors observed strong gains (84 ± 10%) in students’ skills and knowledge over the course of the program (Supplementary Figure S5), and all mentors reported that publications will result from the project, with the intern as co-author. In overall experience (Supplementary Figure S4), students regarded the U-Hack Med Gap Year as very satisfactory and beneficial to their future academic studies (93 ± 9%), and all students would recommend this type of virtual internship and the Department of Bioinformatics as a place for such an internship to others.

All ten students reported that the internship had a definite positive impact on their career plans, and half of the students indicated that applying theoretical concepts to a “real-world” problem was the most satisfying aspect of their internship. For three students the experience solidified their desire to pursue a graduate degree, five indicated a desire to pursue computational biology and/or bioinformatics as a focus of study, and one felt reaffirmed in their decision to pursue a career in industry. Students found that the experience helped to buffer the negative impacts of the pandemic on their career motivation, with one commenting: “I was really struggling to be inspired before I started this internship because of the pandemic and feeling like I was not doing anything, and this really helped to revitalize my love of academia and research.” Another commented, “This internship was incredible both in a professional and personal context. It gave me confidence that I was employable and also that I am good at what I do.”

In open-ended comments, mentors reported that the internship was a very enriching and successful experience, and that they improved their skills in student research mentoring. In comparison with previous undergraduate student mentoring experiences, one mentor felt the length of the internship and the stipend students received resulted in both the mentor and the mentee being “more disciplined and responsive,” and asserted that “as a result, mentors and mentees both gain a more solid experience.”

## Discussion

The program described herein has been a unique opportunity for high-performing students to gain insights into the practices of cutting-edge research in computational biology and medical informatics. The students and mentors expressed their enthusiasm in outcomes surveys and all participating students would recommend this virtual internship and our Department to others. In the future, when COVID-19 restrictions are lifted to allow the in-person U-Hack Med hackathon format again, both events could be organized in tandem, potentially offering hackathon winners a paid virtual internship and/or offering virtual interns the opportunity to serve as team leads for future hackathon projects. The hackathon thereby will enable many students to gain brief insight into computational biomedical research, while the virtual internship will provide an opportunity for exceptional students to work in-depth, longer-term on a dedicated research project in a one-to-one mentorship setting.

For future computational biology internships, we recognized the importance of an early, in-depth training in accessing and using the high-performance computing cluster resources. Such training could be organized, together with other fundamental computing/programming concepts, as “boot-camp” style training that spans the entire first week, instead of a short introduction. A group boot-camp at the beginning of the program may also help build a sense of community among all interns in the cohort. Additionally, more social networking and community building during the internship period was desired by interns, especially as the COVID-19 pandemic had restricted other social interactions.

From the outcomes surveys, we learned that the accompanying biweekly lab meetings provided interns an important framework for professional skills development and reporting on research progress milestones. The meetings can be improved in future programs by stimulating more frequent discussion and constructive feedback from mentors. Furthermore, through exposure to different projects and increased mentor engagement in the meetings, students will be better equipped to make informed choices on academic and career paths, for example, when choosing a suitable topic and a good laboratory for a successful doctoral thesis project.

In addition to the students, mentors benefitted tremendously from the experience. The Department leadership allowed complete freedom to mentors to realize, together with the mentee, a clearly defined research goal. Moreover, the mentors often were trainees (graduate students or postdoctoral fellows), who had fewer prior experiences with research mentoring of an undergraduate student. For these mentors, the U-Hack Med Gap Year program was a supportive environment to grow their mentoring skills, with engaged faculty to advise and guide their mentoring as needed.

Together, in their 9-month U-Hack Med Gap Year internship, students achieved progress toward their research goals and mastered new technical skills, often with little previous knowledge of their subject matter. Fundamental to this process were close mentoring, being part of a cohort, and having a regularly scheduled, shared U-Hack Med Gap Year lab meeting. Overall, the internship was successful in contributing to the students’ and mentors’ academic and career advancement.

## Data Availability

The original contributions presented in the study are included in the article/Supplementary Material, further inquiries can be directed to the corresponding author.
