# Supplementary material for: U-Hack Med Gap Year—A Virtual Undergraduate Internship Program in Computer-Assisted Healthcare and Biomedical Research
Source: Front Bioinform. 2021 Oct 11;1:727066. doi: 10.3389/fbinf.2021.727066 (PMC9581059; doi:10.3389/fbinf.2021.727066)
Supplement: Supplementary file 1 [file DataSheet1.PDF]

Supplementary Material

1 Supplementary Note 1: Student survey results

All participating students (n=10) completed a survey about their experience with the U-Hack Med Gap Year program. For display, we grouped responses into four sections: questions relating the application process (Supplementary Figure 1), bi-weekly lab meetings (Supplementary Figure 2), communication between mentor and student (Supplementary Figure 3), and overall perceptions (Supplementary Figure 4).

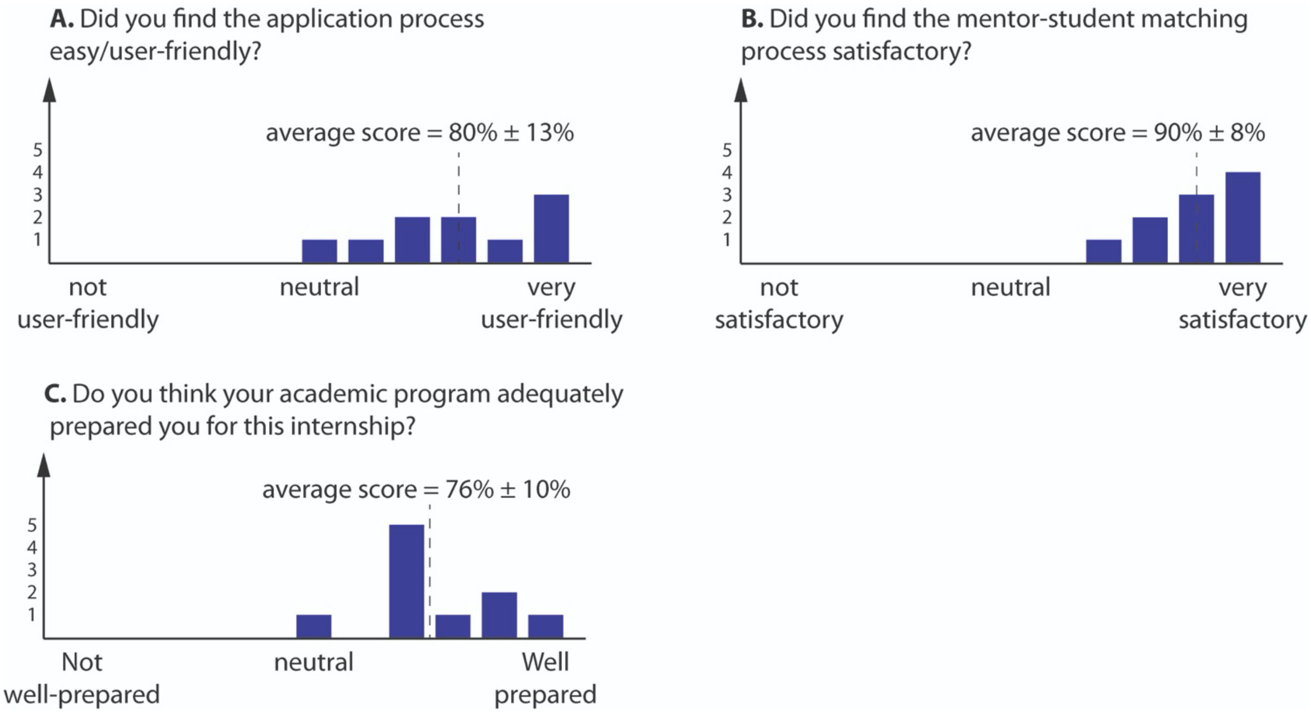

Supplementary Figure 1. Survey results regarding (A, B) the application process and the (C) interns' background. The average score is indicated as a percentage of the optimal value with the 95% confidence interval (student t-distribution). N=10 (all participants).

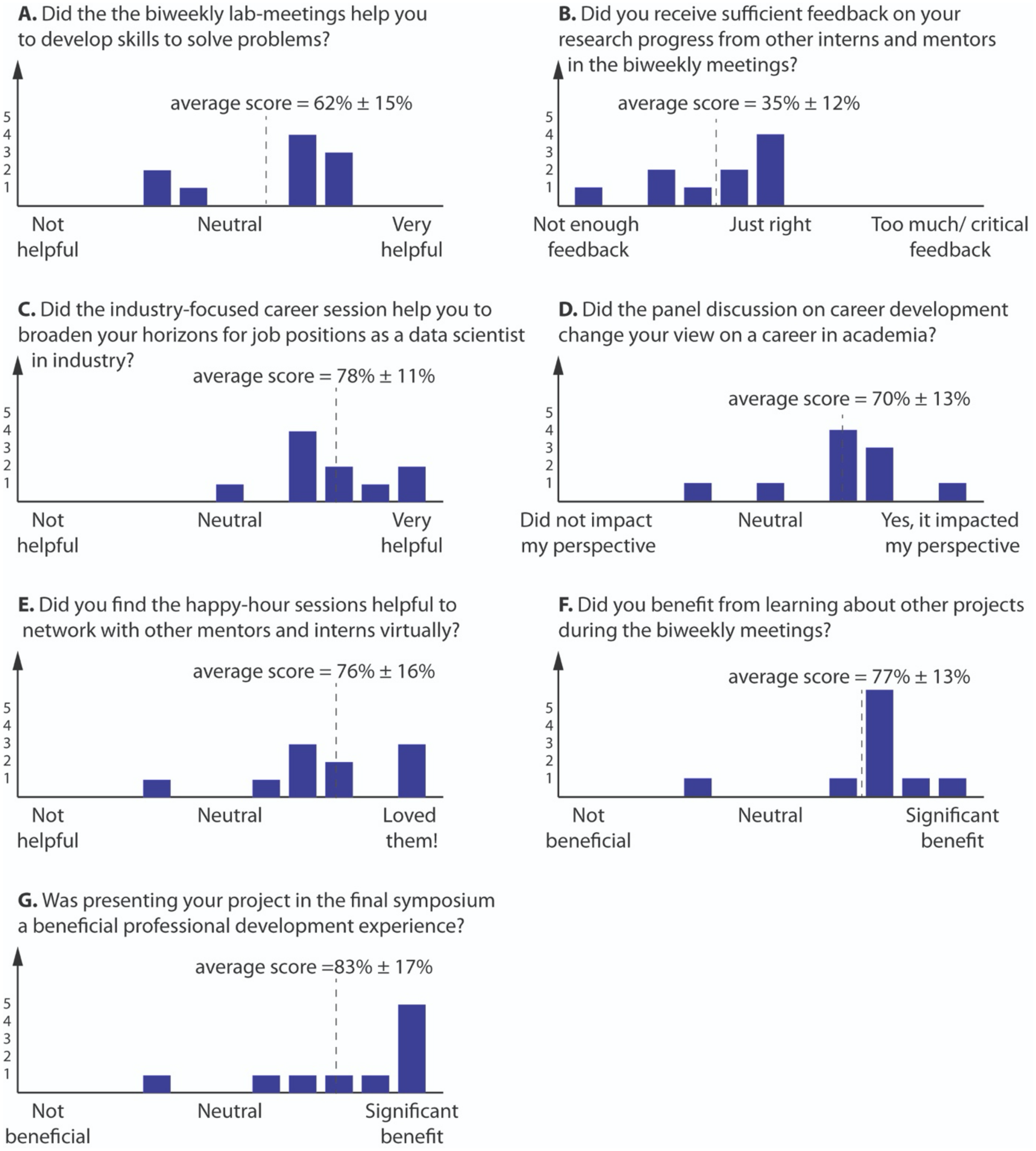

Supplementary Figure 2. Survey results regarding the bi-weekly lab meetings and social networking events. The average score is indicated as a percentage of the optimal value with the student t-distribution 95% confidence interval. N=10 (all participants).

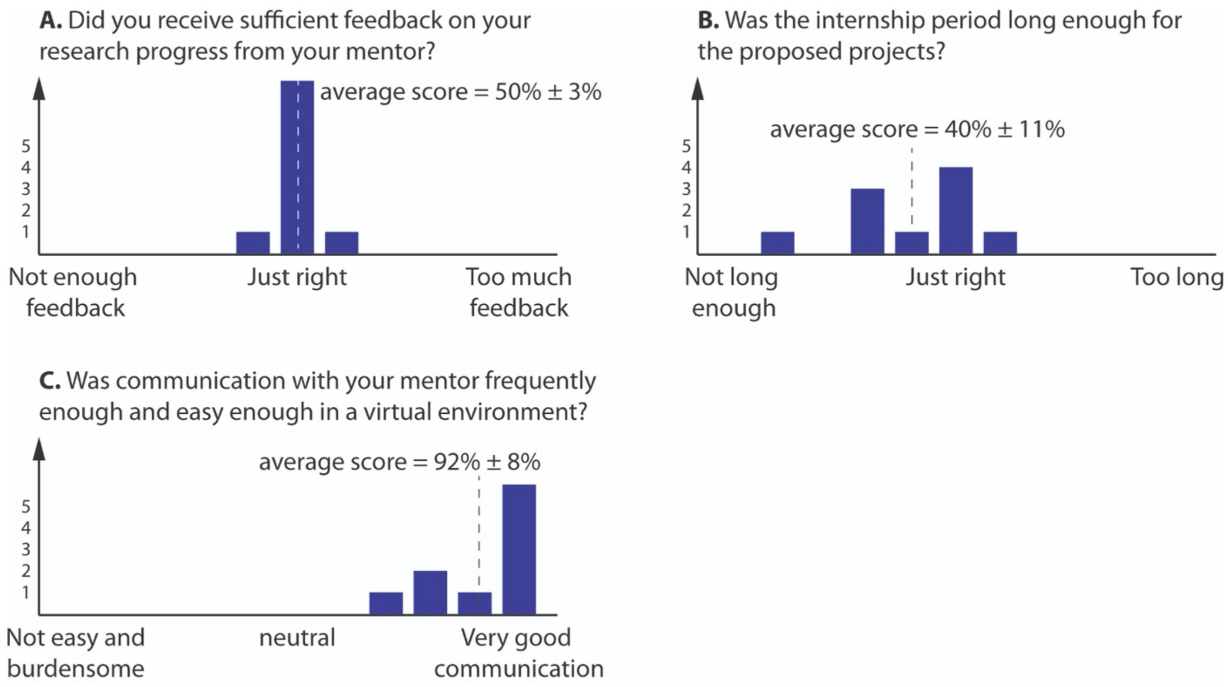

Supplementary Figure 3. Survey results regarding communication between mentor and student, and project duration. The average score is indicated as a percentage of the optimal value with the student t-distribution 95% confidence interval. N=10 (all participants).

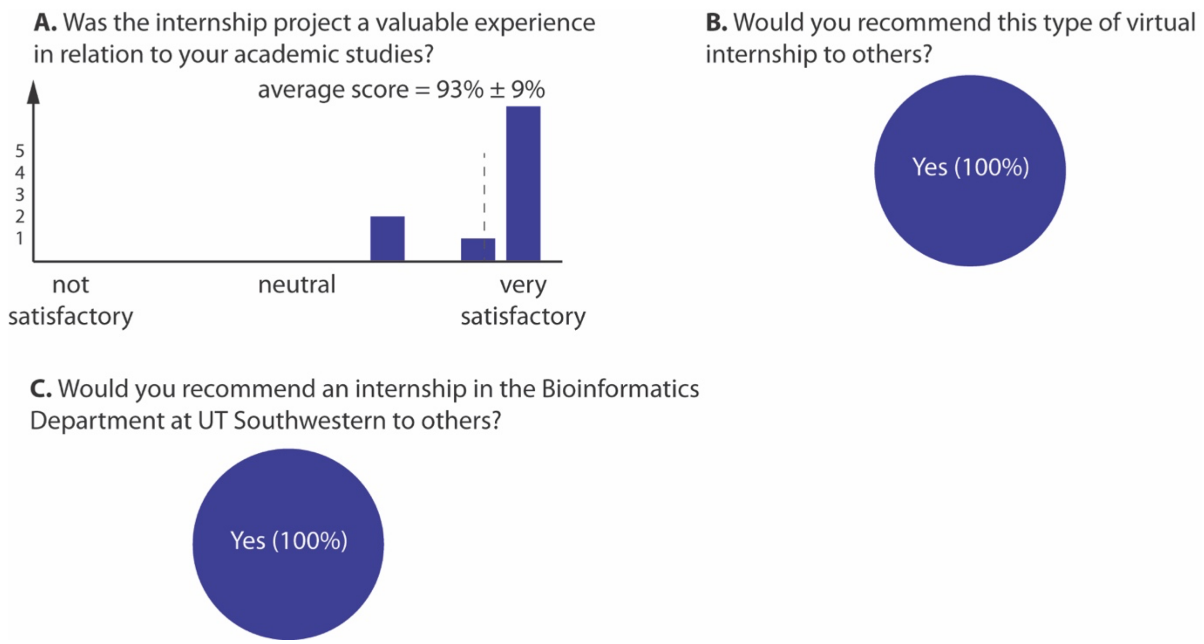

Supplementary Figure 4. Survey results regarding overall perceptions. The average score is indicated as a percentage of the optimal value with the student t-distribution 95% confidence interval. N=10 (all participants).

2 Supplementary Note 2: Mentor survey results

Eight mentors completed a survey on their experience with the U-Hack Med Gap Year program (Supplementary Figure 5).

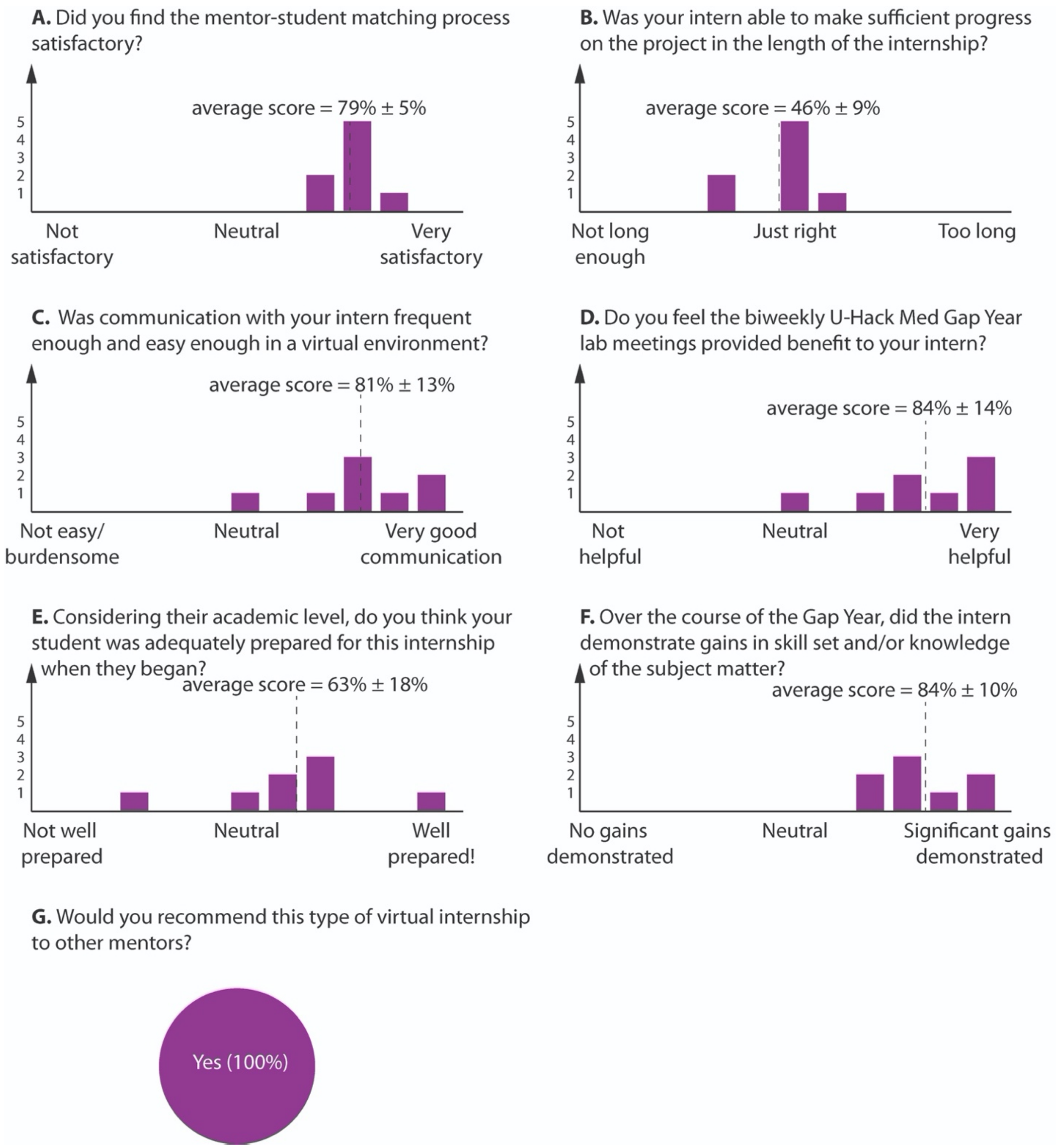

Supplementary Figure 5. Results of mentor survey. The average score is indicated as a percentage of the optimal value with the student t-distribution 95% confidence interval. N=8.
